# How Thailand's greater convergence created sustainable funding for emerging health priorities caused by globalization

**DOI:** 10.3402/gha.v8.28630

**Published:** 2015-08-31

**Authors:** Naowarut Charoenca, Nipapun Kungskulniti, Jeremiah Mock, Stephen Hamann, Prakit Vathesatogkit

**Affiliations:** 1 Faculty of Public Health, Mahidol University, Bangkok, Thailand; 2 Center of Excellence on Environmental Health and Toxicology, Bangkok, Thailand; 3 Center for the Study of Communication-Design, Osaka University, Osaka, Japan; 4 Tobacco Control Research and Knowledge Management Center, Mahidol University, Bangkok, Thailand; 5 Action on Smoking and Health, Bangkok, Thailand

**Keywords:** post-2015 sustainable development goals, globalization, tobacco control, road accidents, prevention, innovative funding, non-communicable diseases, Thailand, low- and middle-income countries

## Abstract

**Background:**

Global health is shifting gradually from a limited focus on individual communicable disease goals to the formulation of broader sustainable health development goals. A major impediment to this shift is that most low- and middle-income countries (LMICs) have not established adequate sustainable funding for health promotion and health infrastructure.

**Objective:**

In this article, we analyze how Thailand, a middle-income country, created a mechanism for sustainable funding for health.

**Design:**

We analyzed the progression of tobacco control and health promotion policies over the past three decades within the wider political-economic and sociocultural context. We constructed a parallel longitudinal analysis of statistical data on one emerging priority – road accidents – to determine whether policy shifts resulted in reduced injuries, hospitalizations and deaths.

**Results:**

In Thailand, the convergence of priorities among national interest groups for sustainable health development created an opportunity to use domestic tax policy and to create a semi-autonomous foundation (ThaiHealth) to address a range of pressing health priorities, including programs that substantially reduced road accidents.

**Conclusions:**

Thailand's strategic process to develop a domestic mechanism for sustainable funding for health may provide LMICs with a roadmap to address emerging health priorities, especially those caused by modernization and globalization.

One of the greatest challenges for improving the health of populations throughout the world is establishing consistent funding (1). For poor and working-class people, particularly in low- and middle-income countries (LMICs), the field of global health holds perhaps the greatest potential for improving their lives (2). Improving a population's health requires resources for research, policymaking, and action. Health leaders and activists call repeatedly for greater integration of health into all aspects of government budgeting (efficiencies) and more accountability (demonstrated impact and effectiveness) (3, 4). Politicians respond again and again by asking for examples of proven ways to create sustainable funding (5).

Promoting and improving health requires establishing funding mechanisms that generate long-term sustainable budget resources (6). The WHO Global Non-communicable Disease Action Plan 2013–2020 and The Lancet Commission on Investing in Health recently identified tobacco taxation as ‘the single most important opportunity for national governments worldwide to curb NCDs because ‘taxation on unhealthy products has the dual benefit of improving the health of the population through reduced consumption, while raising more funds’ (7).

We would argue that in addition to focusing on reducing the use of unhealthy products, governments in LMICs need to focus on establishing consistent funding for emerging pressing problems associated with modernization and globalization (8). Road accidents and injuries are one example, rising rapidly to become the eighth leading cause of death globally, killing an estimated 1.24 million people a year, with 92% of deaths in LMICs (9). Among those who survive road accidents, some 50 million are also injured annually with many suffering lifelong disabilities. Injuries from road accidents especially impact children and young people, being the number one cause of death for children over 5. Unfortunately, the burden from road accidents and injuries is growing with alarming predictions that by 2020 they could be the fifth leading cause of death and disability worldwide (9).

This extraordinary burden in LMICs has resulted in WHO declaring a Decade of Action for Road Safety, 2011–2020, and Bloomberg Philanthropies investing US$125 million over 5 years to promote road safety in 10 high priority countries, mostly LMICs. Bloomberg Philanthropies' investment has been extended in 2015 to include funds to 5 countries and 10 cities worldwide (10). These initiatives have raised some awareness about the importance of road safety and injury prevention, a previously neglected public health area. Experts know that while basic measures of prevention and enforcement can have immediate impacts, other broader factors such as overall national transport, energy, and housing policies are crucial to long-term progress (11). Thus, sustaining national legislation and programs to prevent road accidents, injuries, and death must become a priority in LMICs (12).

Accordingly, we ask the question, can successes in tobacco control be used as an example for people in LMICs to address emerging pressing health development challenges? To answer this question, we looked at the case of how tobacco control in Thailand evolved into consistent funding for broader pressing problems, particularly prevention of road accidents as an example. We conducted a historical review of how Thai health leaders, politicians, and activists achieved sustainable funding for health promotion and disease prevention. Below, we describe the results of our analysis in a narrative form because narratives have face validity that other forms of analysis sometimes lack. For policymakers, narratives are easier to digest, with greater salience and less chance of being dismissed as just hypothetical statistical estimates of ‘harms avoided’ (13).

In this article, we present ideas about how LMICs can create sustainable funding for the prevention of road accidents and injuries based on the experience of building up tobacco control. What follows is an analysis of the evolution of thinking in Thailand that may help LMICs create sustainable funding to prevent an unnecessary health burden of modernization and globalization. We present concrete results that come from establishing a sustainable domestic funding mechanism for health promotion. We highlight this to show an option that may be available to LMICs to improve prevention of emerging health problems, including road accidents and injuries.

## Background

In most countries, resources have been woefully inadequate for health promotion, tobacco control, and the reduction of alcohol consumption. Prevention of accidents and injuries scarcely receives any attention (14). Although global health leaders have called for sustainable funding for health through agreements such as the Monterrey Consensus, and agencies like the WHO have called for individual member states to provide adequate funding for health services and to build health infrastructure, these efforts often have not been successful (15, 16).

Tobacco control funding is a case in point. The World Bank classifies countries as low income, middle income, and high income based on estimated yearly gross national income (GNI) per person. Low-income countries are those with incomes below US$1,045 and include countries such as Bangladesh, Cambodia, and Kenya. Middle-income countries are below US$12,736 and include both lower-middle (US$1,045 to <US$4,125) and upper-middle income (US$4,125 to <US$12,736) countries such as India and Indonesia (lower middle) and Thailand, China, and Brazil (upper middle) (17). In high-income countries, tobacco revenues are 123 times tobacco control spending. In middle-income countries, they are more than 10 times more unequal, with revenues 1,354 times greater than resources spent on tobacco control. Low-income countries spend almost nothing on tobacco control, with revenue from tobacco 54,000+ times what is spent on tobacco control (18). Tobacco control is also resource poor in terms of money from donor contributions for research and programs. Although tobacco use is the only risk factor common to all four leading noncommunicable diseases (NCDs), ‘tobacco control received less than 0.1% of total health-related development assistance in 2007. In 2009, the DACT [Development Assistance to Tobacco Control] per person was 13 and 90 times less than the amount recommended by WHO (US$0.10–$0.72) to control demand for tobacco in LMICs’ (19). Given the current inadequate, unstable funding for tobacco control for LMICs, is there any real opportunity to address the lack of funding?

### The need for innovative funding for emerging health priorities

It is clear that global coordination of funding is vital in laying out principles and plans for providing adequate funding for health. The Monterrey Consensus of 2002 is an important example of such a framework. Some efforts continue with sustainable development goals for mobilizing donors to provide official development assistance. Yet, clearly many goals in the Consensus have not been met and future goals could also fall short depending on political and economic factors. Therefore, many global funding agencies, recognizing that domestic funding is many times greater than donor assistance, have called for innovative national funding approaches. Proposed innovative funding mechanisms for LMICs have included micro-contributions for small projects, public–private partnerships for specialized aid for poverty alleviation and immediate disease challenges, and funding through market-based financial transactions (20). Using exisiting tax policy is perhaps the most accepted and stable form of financial governance. For example, if universal health care is to succeed, universal systems to pay for it will be required if they do not already exist. Are taxes, which are almost as hated in some countries as mandatory insurance programs for health, viable alternatives? The answer depends on what kind and level of tax one proposes. The most acceptable taxes are often specific excise taxes on harmful products or industries like taxes on tobacco and alcohol. These are often called ‘sin taxes’ (21). Have ‘sin taxes’ been successfully used to address national health problems in LMIC?

## Analysis

### The experience in Thailand

The evolution of thinking and action in Thailand provides an example of how a middle-income country, even in the midst of coping with a severe economic crisis, dealt with the need for sustainable funding. In the late 1980s, Thailand had begun to deal seriously with the tobacco epidemic when the US tobacco companies pushed hard to open new markets in Japan, South Korea, Taiwan, and Thailand. At that time, the US Trade Representative, using the threat of trade retaliation, forced open the markets in all the Asian markets mentioned except Thailand. Thailand declined to accede to US pressure, resulting in negotiations and a decision under the General Agreement on Tariffs and Trade, now enforced by the World Trade Organization. Although Thailand was eventually forced to open its market to US-produced cigarettes, the controversy put Thailand squarely among nations that put health before trade and resulted in two comprehensive tobacco control laws that were quite advanced for Asia in 1992. At the time, a few activists were pushing for stronger tobacco control action, but they faced a lack of sustainable funding. Resources from the Ministry of Public Health and the one major nongovernmental organization working on tobacco control, ASH Thailand, provided limited opportunities to develop and deliver tobacco control services and launch interventions. Although the Thai Government adopted a ‘tax for health’ policy to continually raise taxes to discourage smoking, these taxes went into the general fund with little revenue going to tobacco control efforts. The lack of adequate resources caused tobacco control advocates to explore innovations in health funding, including tax mechanisms and the idea of using a portion of tobacco tax revenue to fund tobacco control. At the same time, government officials were considering ways to support the funding of programs addressing the health needs of the poor to show how the government was meeting its responsibility for social equality and economic growth.

From 1995 to 2001, advocates involved in systems research and tobacco control developed a legislative framework consistent with the Ministry of Finance's plans to introduce universal health care. One of the recognized ways to reduce health care costs is to use preventive and health promotion measures to limit costs. Tobacco control, along with many other neglected health promotion areas, fits into the imperative to reduce costs and save lives. Thus, both universal health care and health promotion legislation were passed to provide fuller coverage to everyone and at the same time to lower health care costs through health promotion measures designed primarily to reduce growing morbidity and mortality from tobacco, alcohol, and road accidents (22).

In 2001, Parliament passed the Thai Health Promotion Fund Law establishing the Thai Health Promotion Foundation (ThaiHealth). Reviews of the accomplishments of ThaiHealth at both 5 years (2006) and 10 years (2011) show that ThaiHealth has been very successful (23, 24). Thailand's tobacco control efforts have made it one of the top achievers in meeting the provisions of the first global treaty on health, the Framework Convention on Tobacco Control (FCTC), which was adopted in 2003 and went into effect in 2005 (25, 26). Thailand has taken the lead in encouraging health promotion efforts through its hosting of two global health promotion conferences in 2006 and 2013 and Thailand has been recognized for its overall success with its Universal Health Care scheme. The World Bank in a recent message supporting universal health care displayed an infographic which notes that, ‘Thailand saw more than a 90% decrease over a decade in households impoverished from paying for health care’ (27). Economist Amartya Sen recently noted, ‘The result of universal health coverage in Thailand has been a significant fall in mortality (particularly infant and child mortality, with infant mortality as low as 11 per 1,000) and a remarkable rise in life expectancy, which is now more than 74 years at birth – major achievements for a poor country. There has also been an astonishing removal of historic disparities in infant mortality between the poorer and richer regions of Thailand ….’ (28).

Efforts in tobacco control can reduce global health inequities, and tax policy is vital to both reduce smoking through increasing tobacco prices and as a mechanism to boost tobacco control funding (29). Thailand was recently praised for increasing tobacco prices 230% over 15 years through eight increases in tobacco taxes resulting in a US$6 billion increase in revenue and 4 million fewer smokers than projected based on a smoking rate without tobacco control policies, especially tax increases (30). ThaiHealth provides an example of innovative funding that WHO has used many times to illustrate how funding for NCDs could be accomplished (31). The International Network for Health Promotion Foundations also has highlighted Thailand's approach of using a 2% surcharge based on excise taxes levied on tobacco and alcohol producers and importers to fund health promotion. In Thailand's case, this has produced over US$100 million annually for health promotion efforts, including tobacco control, in Thailand (6).

**Fig. 1:**
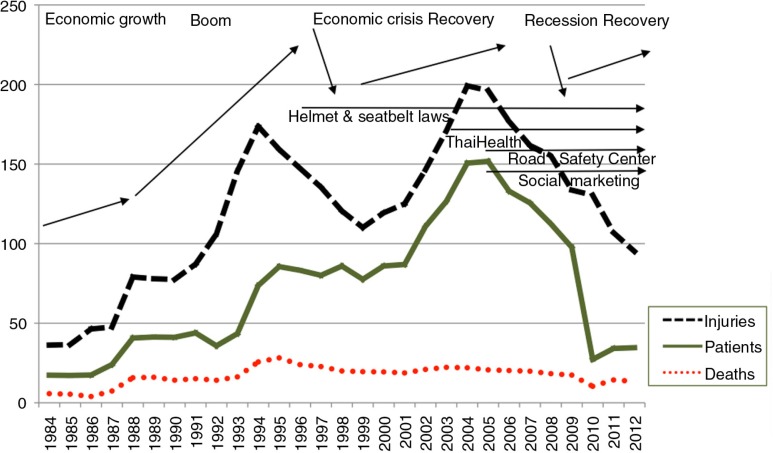
Thailand annual road accident injuries, patients and deaths per 100,000 population, 1984–2012. *Source:* Health Information System Development Office 2015, Royal Thai Police.

While Thailand was achieving substantial reductions in tobacco use, another phenomenon caused by Thailand's rapid modernization and integration into the global economy was injuring and killing Thais in increasing numbers – road accidents (Fig. 1) (32). In the period of Thailand's rapid economic growth from 1984 to 1992, the annual rate of injuries caused by road accidents rose from 36 to 174 per 100,000 population, a 483% increase. Over the same period, cases per 100,000 of hospitalizations from road accidents rose from 17 to 74 (435%), and deaths rose from 6 to 26 (433%). In the years from 1993 to 1999, some investments in road infrastructure appear to have improved conditions, along with the impact of the economic crisis that struck in 1997. However, as the economy recovered, the rising trend returned. By 2004, injuries had reached a new peak of 199 per 100,000 population (a 552% increase over the rate in 1984). Hospitalizations rose to 151 (888%) and deaths to 22 (366%) per 100,000.

Beginning in 2000, a greater convergence of opinion and commitment to action to prevent road accidents and injuries began to emerge in Thailand. ThaiHealth established priorities that included using taxes on tobacco and alcohol to fund prevention programs to reduce road accidents and injuries. ThaiHealth began providing substantial consistent funding year after year to support prevention initiatives with hundreds of partner organizations, including the ThaiRoads Foundation. Between 2001 and 2010, ThaiHealth spent over US$45 million on road-accident-related programs including programs to increase motorcycle helmet use, designate safer school zones, reduce drunk driving, increase enforcement, and establish Thailand's Road Safety Center in 2003.

These and other efforts to strengthen laws on drinking along with the support of a network of organizations with over half a million individual members have resulted in a substantial reduction in road accidents and deaths (32–34). In the period 2004–2012, injuries decreased from 199 per 100,000 to 95, a 52% reduction from the peak rate achieved in just 8 years. Hospitalizations dropped by 77% from 151 to just 35. Deaths dropped by 41% from 22 to 13. Recent evidence of the social return on investment for ThaiHealth actions on road safety showed a 130 times return on investment for funding of interventions of enforcement and prevention. ThaiHealth acts as a catalyst for government and non-government action on road safety. This has provided the basis for the Safer Roads Foundation in 2014 and Bloomberg Philanthropies in 2015 to commit both money and expertise to Thailand to further efforts to significantly reduce road accidents, injuries and deaths and the estimated US$7 billion annual burden from road accidents (34).

## Recommendations

Policymakers and activists should ask themselves, should our goal be to establish a fund for the prevention of road accidents and injuries or a general health promotion fund? In countries that do not have either type of fund, the decision about which type of fund to establish should be based on a careful assessment of the range and depth of public support and political feasibility to establish a fund with the broadest possible mandate. In Thailand's case, data on road accidents and alcohol-related problems substantially influenced politicians to support the establishment of a general health promotion fund. We recommend establishing a general health promotion fund with a broad mandate that includes support for road accident and injury prevention projects. In LMICs that already have a health promotion fund, we recommend that the fund should support road accident prevention programs.

It is important to investigate different mechanisms for establishing sustainable domestic funding for emerging health priorities. Based on our analysis of Thailand's experience in establishing a general health promotion fund (ThaiHealth), we identified the following potential mechanisms that could be used to establish either a general health promotion fund or a specific fund for preventing road accidents.

### Tobacco and Alcohol

Since tobacco and alcohol consumption contribute significantly to road accidents and injuries in most countries, it is logical to argue for a combination of specific and ad valorem excise taxes on tobacco products and alcoholic beverages that is earmarked specifically to fund health promotion or road accident and injury prevention programs.

### Fuel tax

An excise tax on vehicle fuel is also a good potential source of revenue. Fuel consumption is closely correlated with vehicle use, so when use increases and accident risk increases, revenue also increases. Many countries, including Thailand, already have a ‘gasoline fund’ taken from a portion of gasoline tax revenue, but this fund should not be used to fund health programs because it is designed to mitigate against sudden increases in oil prices. Such increases could require the government to tap into reserves in a gasoline fund, thus stripping funding away from a health promotion or accident prevention fund.

### Vehicle-related taxes

These include four possibilities: 1) an ad valorem excise tax on vehicle purchases, 2) an annual registration fee with an earmarked surcharge, 3) a vehicle insurance fee, and 4) a portion of fines from traffic law violations.

In deciding which funding source or sources to use, the following criteria should be considered: ease of collection with low transaction costs, a sufficient, stable revenue stream to serve the purpose, sustainability of the source and the political feasibility of using the source for road accident and injury prevention. Sometimes using a surcharge or additional tax is the key to political acceptability. Whatever the intended source of funding, it should be ‘new money’, not funds deducted from an existing revenue stream.

It is important to anticipate obstacles that may arise in advocating to establish a new fund. Our analysis of Thailand's experience establishing ThaiHealth shows that some obstacles may emerge: 1) the Ministry of Finance may want funds to come from the regular budget not from a dedicated source, 2) the government may want the fund to be managed within the existing administrative system, not by an autonomous agency, 3) the government may want the fund to be established through an executive order, not through new legislation, 4) different ministries may fight to be in charge of the fund, and 5) there may be opposition from affected industries.

Evidence-based planning is essential for generating political support and mobilizing resources. Operational research will probably be needed to support advocacy to set up a new fund. Since prevention of road accidents and injuries is an emerging problem in countries with limited resources for health, country-specific research should include: 1) the nature and burden of road accidents, 2) their economic consequences, including costs and potential savings from preventing those costs, 3) identification of issues related to the proposed fund itself including the source and operation of the fund, and specific details on the governance of the fund. The latter should include pros and cons of a fund under an independent statutory agency or as a unit of an existing government department.

If planners decide to advocate for establishing a general health promotion fund that will also fund prevention of road accidents, research on NCDs should be gathered and presented as a comprehensive package with accident prevention. Advocating for a broad-based health promotion fund that includes many neglected priorities is likely to receive more support from politicians with diverse interests and constituencies.

It is also important to anticipate obstacles that may arise after establishing a fund. Below are some possible obstacles administrators may face after establishing a fund:

1. The ‘happiness problem’, that is, having the money, but not knowing how to best use it, or not being able to allocate it fast enough.
2. Inadequate human resources and technical know-how to design and administer prevention projects.
3. Inadequate absorptive capacity to disburse the funds due to lack of good projects, lack of organizations or partners to run projects, particularly community-based participatory projects.
4. Inadequate transparency, risks of abuse and corruption, and political interference.

To prevent these potential problems, the founding documents that establish the fund in legislation or otherwise should specify the scope and mission of the fund, and the governance structure of the fund. We recommend that a fund be governed by an independent board, with both public and academic experts included on the board. Furthermore, founding documents should specify the nature of projects entitled to consideration for funding and limit the fund to supporting project-based activities. Strategies for using innovative and sustainable sources of funding to address emerging health priorities should include short-, medium-, and long-term targets. However, starting with small and achievable targets is advisable.

## Summary

Based on experience in Thailand, key steps in a process to establish sustainable funding for emerging health development priorities, including health promotion, tobacco and alcohol control, and the prevention of road accidents and injuries are:

1. Setting up a focal point to work on establishing a fund.
2. Creating a multi-sectorial coalition of partners.
3. Creating a national network of organizations and individuals.
4. Designating a passionate person to lead the advocacy.
5. Advocating to draw up a national plan.
6. Identifying a political champion who will support establishing a fund.
7. Recruiting victims and their families to be allies.
8. Organizing forums to advocate for a sustainable funding mechanism.
9. Publicizing health problems through a range of media channels.
10. Generating research with useful data.
11. Proactively monitoring health problems through surveillance.
12. Practicing positive thinking and perseverance in efforts.

This is a challenging agenda that will take time to fulfill. Some measures can be undertaken quickly with limited funding. Media advocacy (items 8 and 9) is not very costly, but it can be highly effective in reaching the public and in creating political awareness, which in turn, can produce increasing political commitment. Analysis of the programs needed (items 10 and 11) can also be undertaken with limited funding.

## Policy implications

Nearly 25 years ago, one senior author of this article warned that a tobacco epidemic would likely spread throughout Asia (35). The initial experience in Thailand showed how a tobacco epidemic caused by globalization can spread when there are inadequate resources and a lack of infrastructure to deal with it. Thailand's response through tobacco control and health promotion provides an example of how an epidemic can be averted. In addition, Thailand's approach to addressing insufficiencies in funding may be a useful example for LMICs. Thai advocates and policymakers recognized a need for sustainable funding and sought to establish a domestic mechanism to provide long-term resources (36).

In this era of globalization, an enduring improvement in people's quality of life will only be achieved through sufficiency in areas of human development. Thailand's once insufficient control of the globalized tobacco industry gave rise to a greater convergence between health advocates, politicians and other members of Thai society. The convergence emerged because those concerned managed to see past their competing self-interests. The greater convergence presented here is a convergence that has come through health and governance movements that have now come into focus and will likely continue to propel our efforts through the coming century.

The example of establishing a health promotion fund with a mandate to prevent road accidents and injuries shows how preparing evidence, using strategies sensitive to political realities, and forging relationships for innovation can result in success. This is not a fail-safe formula, but a reasonable long-term approach that can have impacts of great importance. Domestic, sustainable funding for health projects and services can produce big contributions toward achieving sustainable development goals.

For most LMICs, the increasing rates of road accidents and injuries have reached a crisis level. Therefore, investing resources in this area requires immediate action (37). In addition, many other health development priorities remain neglected because of a lack of funding. The prospects of improved health through supranational organizations supporting sustainable development are emerging. Prevention of road accidents and injuries needs to be at the forefront of the agenda. Donor aid will likely be committed for the push to eliminate poverty and establish social, economic, and environmental sustainability for all. These priorities are important, but depending on supranational organizations for funding perpetuates dependency and programmatic instability. Thus, to ensure sustainability, LMICs must establish their own innovative funding initiatives to grapple with the surging negative effects of modernization and globalization.
